# Sustainable Absorbent Pads from Polybutylene Adipate Terephthalate/Thermoplastic Starch Films Combined with Hairy Basil (*Ocimum basilicum*) Powder to Enhance Meat Shelf Life

**DOI:** 10.3390/foods14091525

**Published:** 2025-04-26

**Authors:** Fuengnapha Khunta, Korakot Charoensri, Rineta Pertiwi Nurhadi, Nattinee Bumbudsanpharoke, Pontree Itkor, Youn-Suk Lee, Athip Boonsiriwit

**Affiliations:** 1 Department of Food Science and Technology, Thammasat University, Pathum Thani 12120, Thailand; fuengnapha.khu@outlook.com; 2 Department of Biotechnology, Korea University, Seoul 02841, Republic of Korea; korapop253@korea.edu; 3 Department of Packaging and Materials Technology, Kasetsart University, Bangkok 10900, Thailand; rinetapertiwi.n@ku.th (R.P.N.); nattinee.bu@ku.ac.th (N.B.); 4 Department of Packaging, Yonsei University, Wonju 26494, Republic of Korea; pontree.itkor@gmail.com (P.I.); leeyouns@yonsei.ac.kr (Y.-S.L.); 5 Center of Excellence in Food Science and Innovation, Thammasat University, Pathum Thani 12120, Thailand

**Keywords:** absorbent pad, biobased materials, hairy basil seed, shelf life, fresh pork preservation

## Abstract

This research developed a biodegradable absorbent pad using polybutylene adipate terephthalate (PBAT) and thermoplastic starch (TPS) films, combined with hairy basil (*Ocimum basilicum*) seed powder (HBP) to extend the shelf life of fresh pork during cold storage. To form the biocomposite film, PBAT was blended with TPS in ratios of 100:0, 90:10, 70:30, and 50:50. The PBAT:TPS ratio of 70:30 (PB7T3) was the most suitable in terms of mechanical properties and water permeation. Therefore, PB7T3 was selected to fabricate the absorbent pad for extending the shelf life of fresh pork during cold storage. For the storage test, 100 g of pork pieces was placed in PET trays (12 cm × 12 cm), each containing a different absorbent: the control (no pad), a commercial absorbent pad, and the PB7T3 absorbent pad. The pork samples were stored at 4 °C for 8 days and analyzed for color change, total plate count (TPC), total volatile basic nitrogen (TVB-N), pH, and drip loss on days 0, 2, 4, 6, and 8. The results indicated that the PB7T3 absorbent pad effectively extended the shelf life of fresh pork compared to the control, with no significant difference compared to the commercial absorbent pad made from plastic. This research opens new avenues for developing sustainable absorbent pads, contributing to reduced reliance on conventional non-biodegradable plastics.

## 1. Introduction

Fresh meat undergoes drip loss during storage. This encourages the growth of microorganisms because fresh meat is rich in nutrients and has high water activity, thereby reducing product shelf life and commercial value [1,2]. To address this issue, packaging technology is used to extend the shelf life of meat products and maintain their quality.

Liquid-absorbent pads, a common solution in meat packaging, effectively address spoilage issues by absorbing excess liquid, such as water and blood. This hinders microbial growth, thereby extending product shelf life and preserving its smell, taste, and texture [3]. Most absorbent pads are made from polyethylene (PE) plastic, which is recyclable [4], but contamination from substances like blood makes the pads difficult to clean and recycle, limiting their sustainability. As a result, these pads are typically disposed of in landfills or incinerated, contributing to environmental pollution. Biodegradable absorbent pads made from agricultural sources present a more environmentally friendly alternative, offering the same functionality while reducing plastic waste.

PBAT (polybutylene adipate terephthalate) is one of the most widely used biodegradable polymers in the production of environmentally friendly products, including shopping bags, agricultural mulch films, garbage bags, and food packaging [5]. It is considered safe for food contact and complies with regulations like EU Regulation (EU) No 10/2011 and FDA requirements [6]. However, due to its relatively high cost, PBAT is often blended with other polymers to reduce production expenses. PBAT/starch blends have gained significant attention as a viable alternative to petroleum-derived polymers, particularly for applications involving direct food contact. Moreover, a film blend of PBAT and starch increases water transmittance [7] and is suitable for making an absorbent pad sachet.

Several natural materials can be used to produce biodegradable alternatives, including polysaccharides [8], lipids [9], and proteins [10], with polysaccharides most commonly used for biodegradable packaging due to their low cost and local availability [11].

Hairy basil seeds (*Ocimum basilicum* L.) are widely used in food and beverages across many cultures and recognized as safe for consumption [12]. In Thailand, they have long been consumed for their nutritional and medicinal benefits. These seeds also show significant potential as a source of biodegradable materials containing cellulose, hemicellulose, and lignin, while their mucilage is rich in pectin, arabinoxylan, glucomannan, and other hemicellulose compounds [13]. These components can be broken down by microbial activity without leaving harmful residues, making hairy basil seeds suitable for developing biodegradable liquid absorbents.

This research developed a sachet for absorbent pads using a blend of PBAT and thermoplastic starch (TPS) at various ratios to determine the optimal formulation. The selected PBAT/TPS sachet was then combined with polymer extracted from hairy basil seeds, which served as the absorbent material inside of the sachet. Finally, the absorbent pad was applied to extend the shelf life of fresh pork during cold storage.

## 2. Materials and Methods

### 2.1. Materials

Polybutylene adipate terephthalate (PBAT, Ecoflex^®^F Blend C1200, melting point 110–120 °C) pellets were purchased from BASF, Ludwigshafen, Germany. Acetylated cassava starch powder (degree of substitution 0.01–0.03, KREATION^®^ SS) from SMS Corp., Pathum Thani, Thailand was used for TPS production. Commercial-grade glycerol was purchased from P. Wai Co., Ltd., Bangkok, Thailand and used as the plasticizer.

### 2.2. Biocomposite Film Preparation and Characterization

#### 2.2.1. Biocomposite Film Preparation

Fabrication of the PBAT/TPS biocomposite film was performed following Bumbudsanpharoke, Nurhadi, Chongcharoenyanon, Kwon, Harnkarnsujarit, and Ko [7]. Acetylated cassava starch powder was pre-dried in an oven at 60 °C overnight. During the extrusion of TPS pellets, 30 wt% glycerol was introduced to the feeding zone of a twin-screw extruder (Labtech Engineering, Samut Prakan, Thailand). Extrusion was carried out with a pre-set heating profile of 90–150 °C and a screw speed of 180 rpm. The extruded TPS was cooled to room temperature and pelletized using a pelletizer (Labtech Engineering, Samut Prakan, Thailand), yielding TPS pellets with a melting temperature range of 150–180 °C. PBAT and TPS pellets were dried at 60 °C overnight and then manually blended in PBAT:TPS ratios of 100:0, 90:10, 70:30, and 50:50. The resulting PBAT/TPS biocomposites were designated as PBAT, PB9T1, PB7T3, and PB5T5, respectively. All compounding steps were continuously performed in the extruder under the same temperature and screw speed conditions as those previously described.

#### 2.2.2. PBAT/TPS Biocomposite Film Characterization

The morphology of the PBAT/TPS biocomposite films was determined through scanning electron microscopy (SEM) (Leica Microsystems Ltd., Cambridge, UK) at an acceleration voltage of 15 kV under high-vacuum conditions. Before imaging, a thin gold layer was sputter-coated onto the fractured surface to prevent charging when exposed to the electron beam.

The chemical structures of the films were characterized using an Attenuated Total Reflection–Fourier Transform Infrared (ATR-FTIR) Spectrometer (1760X, PerkinElmer Life & Analytical Sciences, Inc., Waltham, MA, USA). The samples were scanned over a wavenumber range of 4000 to 400 cm^−1^, with results reported as the average of three replications.

The thickness of each composite film was measured using a digital micrometer (Mitutoyo Manufacturing Co., Kawasaki, Japan). Mechanical properties of the PBAT/TPS biocomposite films were tested on a 3300 Series Universal Testing Machine (Instron Engineering Corp., Norwood, MA, USA) with a load cell of 200 N and a crosshead speed of 500 mm/min with distance between the handles of 5 cm (ASTM D882–18). An average of at least ten replicates was reported for each sample.

The water vapor transmission rate (WVTR) of the PBAT/TPS biocomposite films was measured using a 7000 Water Vapor Permeability Tester (Illinois Instruments, Inc., Johnsburg, IL, USA) at 38 °C and 90% relative humidity, with results presented as water vapor permeability (WVP) at g·mm/m^2^·day·Kpa.

### 2.3. Hairy Basil Seed Powder (HBP) Preparation

Hairy basil seeds were purchased from a local supermarket in Pathum Thani Province. The seeds were cleaned and then ground into powder using a high-speed electric grinder. A total of 100 g of hairy basil seed powder (HBP) was defatted by washing with 500 mL of petroleum ether, repeating the process until the solvent became clear. The defatted powder was then air-dried overnight at room temperature under a fume hood until the moisture content dropped below 15%. The dried HBP was sieved through a 140-mesh screen to obtain a particle size of approximately 100 μm. The final powder was stored in a plastic zip-lock bag and kept in a desiccator at room temperature [14].

### 2.4. Fabrication and Characterization of the Biodegradable Pad

#### 2.4.1. Fabrication of the Absorbent Pad

Each composite film was cut into 8 × 11 cm pieces and sealed on three sides using a heat-sealing machine to form pouches. To match the absorption capacity of the commercial absorbent pad used as a reference in this study, 6 g of HBP was placed into each pouch. The fourth side was then sealed using the same heat-sealing machine to obtain the final absorbent pad.

#### 2.4.2. Determination of Water Absorbing Capacity (WAC)

The water absorption capacity (*WAC*) of the pad was determined following the method described by Lee, et al. [15], with some modifications. First, the initial weight of the dried pad was recorded. The pad was then immersed in 50 mL of deionized (DI) water on a PET tray. The wet pad with the DI water and the volume of the remaining DI water in the PET tray were measured at 1 h intervals up to 96 h. The *WAC* of the pad was calculated using the formula

(1) $$WAC\%=(\frac{W_{w}-W_{b}}{W_{b}})\times 100$$

where *W_w_* is the weight of the wet pad and *W_b_* is the weight of the unabsorbed baseline pad.

### 2.5. Application of the Pad to Chilled Pork Packaging

#### 2.5.1. Chilled Pork Packaging Preparation

Fresh, chilled pork was purchased from a local grocery store and cut into pieces (8 cm × 10 cm), each weighing 100 g. The pork pieces were then placed on PET trays (12 cm × 12 cm), with each tray containing a commercial absorbent pad or a PBAT/TPS biocomposite absorbent pad incorporating HBP. A pork sample without an absorbent pad was used as the control. Each tray was wrapped with PVC film, and 20 boxes were prepared for each treatment. All samples were stored at 4 °C with 80% RH and analyzed on days 0, 2, 4, 6, 8, and 10 of storage.

#### 2.5.2. Measurement of Drip Loss

Drip loss measurement was performed according to Wang, et al. [16]. First, the sample was removed from the tray and weighed. Using the corresponding fresh weight of the beef before packaging, drip loss (%) was calculated as

(2) $$drip\;loss\left(\%\right)=\frac{fresh\;weight-weight\;at\;sampling}{fresh\;weight}\times 100$$

#### 2.5.3. Color Change

A digital camera using a CR-400 Chroma Meter was used to obtain the L*, a*, and b* values of the surface color of the pork in contact with the absorbent pads. The instrument was calibrated with both white and black standard tiles before each measurement.

#### 2.5.4. TVB-N

TVB-N was performed according to Wang, Yan, Xu, Liu, and Chen [16], with some modifications. The pork samples were separately ground, and 10 ± 0.1 g of the ground pork was blended with 100 mL of distilled water in a stomacher bag, followed by homogenization for 5 min at 200 rpm using a stomacher. After filtration, 5 mL of the filtrate was mixed with 5 mL of 10 g/L magnesium oxide (MgO) in a Kjeldahl distillation unit. Steam distillation was performed for 5 min. The distillate was absorbed in 10 mL of 20 g/L boric acid and then titrated with 0.01 mol/L HCl. The total volatile base nitrogen (*TVB-N*) was calculated using the equation

(3) $$TVB-N=\frac{\left(V_{2}-V_{1}\right)\times C\times 14\times f}{W}\times 100$$

where *V*_2_ = volume of HCl consumed by the sample (mL), *V*_1_ = volume of HCl consumed by the blank (mL), *C* = concentration of HCl (M), *W* = sample quantity (g), and *f* = dilution factor. The *TVB-N* content was reported as mg/100 g of pork meat.

#### 2.5.5. Microbial Analysis

Pork samples (25 g) were aseptically mixed with 225 mL of 0.85% sterile saline in a stomacher bag and homogenized at 200 rpm for 2 min. Serial dilutions of the homogenate were prepared in 0.85% sterile saline, and three appropriate dilutions were selected for testing. Microbial analysis was performed using Aerobic Count Petrifilm plates (3M, St. Paul, MN, USA) incubated aerobically at 35 °C for 24 to 48 h [17]. Total plate count (TPC) was calculated and expressed as log10 CFU/g.

#### 2.5.6. pH Measurement

The pH of the pork samples was measured following the method described by Miao, et al. [18]. Triplicate pork samples were homogenized in 90 mL of distilled water and filtered. The pH was then determined using a digital pH meter (AB15pH, Fisher Scientific Co., Waltham, MA, USA).

### 2.6. Statistical Analysis

All of the experiments were conducted at least in triplicate. Experimental data were analyzed using SPSS (SPSS 25 for Windows, SPSS Inc., Chicago, IL, USA) and analysis of variance (ANOVA). Statistical significance of the difference in mean values was established at *p* ≤ 0.05, and Duncan’s new multiple range test was utilized for all statistical analyses.

## 3. Results and Discussion

The PBAT/TPS biocomposite film with 90% PBAT and 10% TPS showed phase separation and unstable tubular during blowing, as displayed in the Supplementary Data. Therefore, the results for PB9T1 were excluded from this report.

### 3.1. Morphology

**Figure 1:**
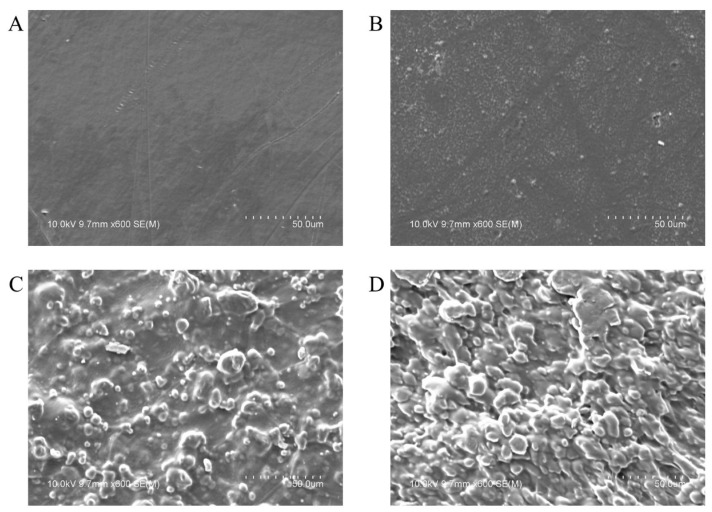
FE-SEM images of the surface of the LDPE (**A**), PBAT (**B**), BA7T3 (**C**), and PB5T5 (**D**) films.

The microstructures of the film surfaces were examined using field-emission scanning electron microscopy (FE-SEM), as shown in Figure 1. The LDPE film (Figure 1A), used as a representative commercial plastic, displayed a smooth and homogeneous surface. Similarly, the PBAT film (Figure 1B) exhibited a uniform structure without pores or major defects. As the TPS concentration increased, visible starch granules became more prominent, as observed in PB7T3 (Figure 1C) and PB5T5 (Figure 1D). However, no cracks or wrinkles were evident across the polymer phases, and the overall film surfaces remained intact. These findings concurred with Zhai, et al. [19].

### 3.2. Mechanical and Barrier Properties

The thickness, mechanical, and barrier properties of packaging materials are some of the most fundamental and important performance indicators when evaluating application performance. The thicknesses of the fabricated films ranged from 28.00 ± 0.90 to 32.20 ± 0.80 μm (Table 1). The incorporation of TPS into PBAT significantly increased the film thickness from 28.40 μm to 32.20 μm with 50% TPS.

Mechanical properties, including tensile strength (TS) and elongation, of the LDPE and PBAT/TPS biocomposite films are presented in Table 1. Tensile strength represents the maximum stress a film can withstand before breaking. The TS of the LDPE film was 11.71 ± 1.13 N, which was approximately 2.5 times lower than that of pure PBAT. As the TPS content increased, the tensile strength of the PBAT/TPS biocomposite films showed a continuous decline, dropping from 29.02 ± 1.26 N to 2.15 ± 1.58 N when 50% TPS was incorporated into the PBAT matrix, representing a 92.59% reduction.

A similar decreasing trend was observed in the elongation of PBAT/TPS biocomposite films as the TPS content increased. Compared to pure PBAT, the elongation of PB7T3 and PB5T5 films decreased by 32% and 89.9%, respectively. This reduction is likely due to the disruption of PBAT’s original continuous polymer network caused by poor compatibility between hydrophilic TPS and hydrophobic PBAT [20], as is also evident in the SEM images shown in Figure 1. However, there was no significant difference in elongation between the LDPE and PB7T3 films, indicating that PB7T3 possesses similar flexibility and tear resistance to the commercial plastic.

The water vapor permeability (WVP) of LDPE was 0.76 ± 0.04 g·mm/m^2^·day·kPa. In comparison, the WVP values for PBAT, PB7T3, and PB5T5 were significantly higher, ranging from 2.76 ± 0.14 to 4.89 ± 0.43 and 5.51 ± 0.24 g·mm/m^2^·day·kPa, respectively. The results showed a clear increase in WVP with rising TPS content. This increase is attributed to the hygroscopic nature of starch, which has a higher affinity for water compared to PBAT, thereby enhancing water vapor permeability in starch-filled films [21]. These results concurred with Bumbudsanpharoke, Nurhadi, Chongcharoenyanon, Kwon, Harnkarnsujarit, and Ko [7] and Phothisarattana, et al. [22], who studied biocomposite PBAT/TPS films.

### 3.3. ATR-FTIR Analysis

**Figure 2:**
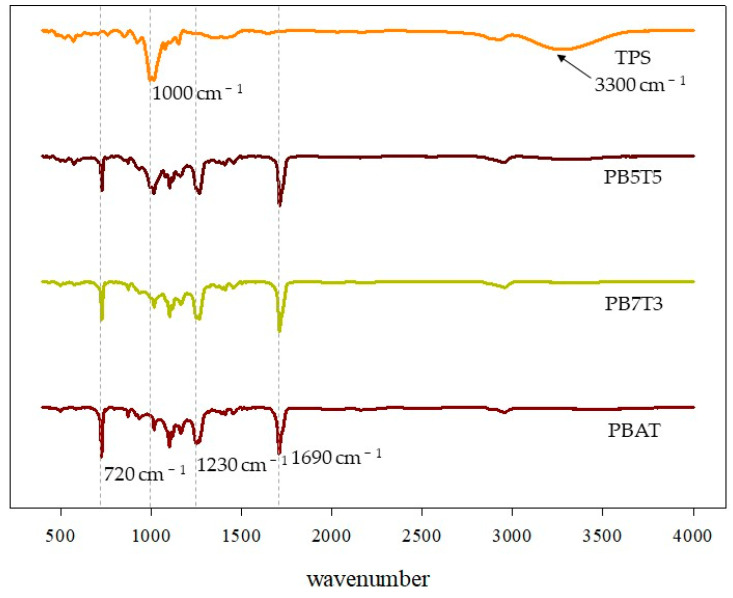
FTIR spectra of PBAT, PB7T3, PB5T5, and TPS.

Infrared (IR) spectra of the samples were obtained using ATR-FTIR spectroscopy to identify specific chemical groups in the biocomposite films. The FTIR spectra of PBAT, PB7T3, PB5T5, and TPS in the range of 400–4000 cm^−1^ are shown in Figure 2. The spectra of PB7T3 and PB5T5 exhibited absorption bands similar to PBAT, with 1690 cm^−1^ corresponding to C=O stretching vibration, 1230 cm^−1^ to C−O stretching vibrations in ester groups, and 720 cm^−1^ to stretching vibrations of multiple adjacent methyl groups [19,22,23]. In TPS, peaks were observed at 3300 cm^−1^ and 1000 cm^−1^, corresponding to O−H stretching vibration and C−O stretching in the glycosidic bond [24], respectively. All PBAT/TPS biocomposite films showed increases in the absorbance bands around 3300 cm^−1^ and 1000 cm^−1^ as the TPS content increased, attributed to the large number of hydroxyl groups in TPS being introduced into the films. These results confirmed that TPS was successfully incorporated into the films.

### 3.4. Characterization of the Absorbent Pads

**Figure 3:**
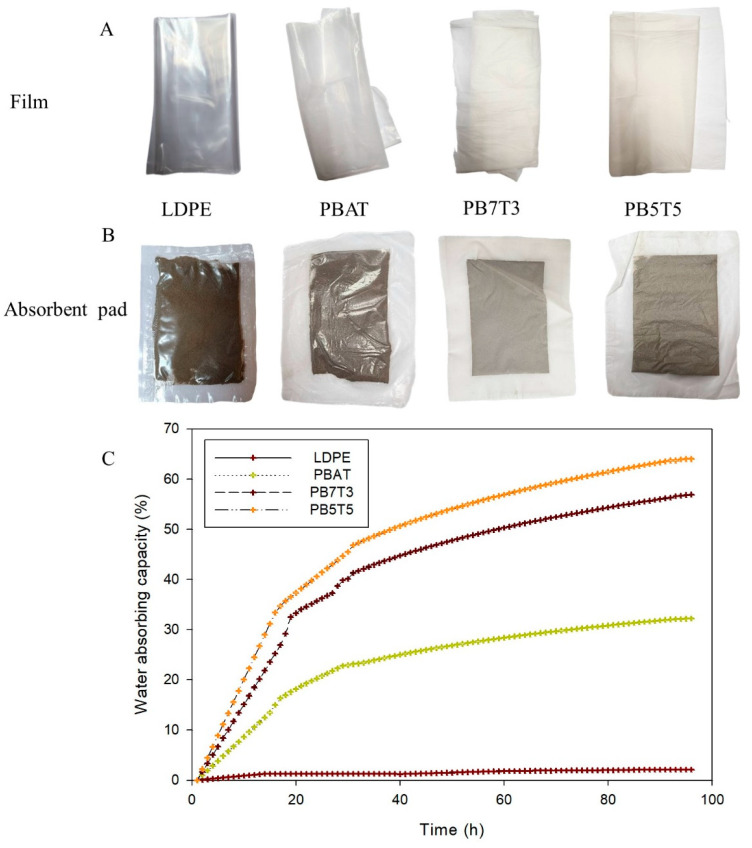
Extruded blown film (**A**), absorbent pad with various materials (**B**), and water absorbing capacity of absorbent pad (**C**).

Film samples and absorbent pads made from different types of films are shown in Figure 3A and Figure 3B, respectively. Absorbent pads are commonly placed under packaged fresh foods, such as meat, poultry, or fish, to absorb excess liquids. The water absorption capacity (WAC) of the LDPE and the developed PBAT/TPS biocomposite were evaluated by immersing the absorbent pads in deionized (DI) water, with the results presented in Figure 3C.

The WAC of LDPE gradually increased from 0% to 1.27% over the first 14 h and then slowly rose to 2.14% after 96 h. In contrast, the WAC of the PBAT, PB7T3, and PB5T5 absorbent pads increased sharply during the first 30 h. After 36 h, the rate of water absorption slowed and approached equilibrium around 90 h. Among the samples, PB5T5 showed the highest WAC, followed by PB7T3 and PBAT. After 96 h of soaking, the WAC values were 64.1% for PB5T5, 56.9% for PB7T3, 32.18% for PBAT, and only 2.14% for LDPE. These results clearly indicate that increasing the TPS content in the PBAT matrix significantly enhances the water absorption capacity of the absorbent pads. This trend is consistent with the WVP results of the films, which also increased with higher TPS content, further supporting the improved water affinity of the biocomposites.

Based on the characterization results of the film and the absorbent pad, PB5T5 exhibited the highest WAC but had the lowest physical properties. During biocomposite processing, PB5T5 also had the lowest yield, and it was challenging to blow into a film. In contrast, the PB7T3 film demonstrated better physical properties, good WAC, easier film-blowing capability, and higher yield. Therefore, PB7T3 was selected for use in fresh pork packaging in the next study. To compare it with the commercial absorbent pad, the PB7T3 pad was perforated with micro pinholes at a surface density of nine holes/cm^2^.

### 3.5. Application of PBAT/TPS Biocomposites in Fresh Pork Packaging

Fresh meat products naturally generate exuded liquid during transportation and handling. The presence of this exudate in plastic meat trays is perceived by customers as a sign of low quality and creates an environment that promotes the growth of spoilage microorganisms and pathogens due to its nutrient-rich composition [16]. To address this issue, absorbent pads are used to manage the excess liquid.

**Figure 4:**
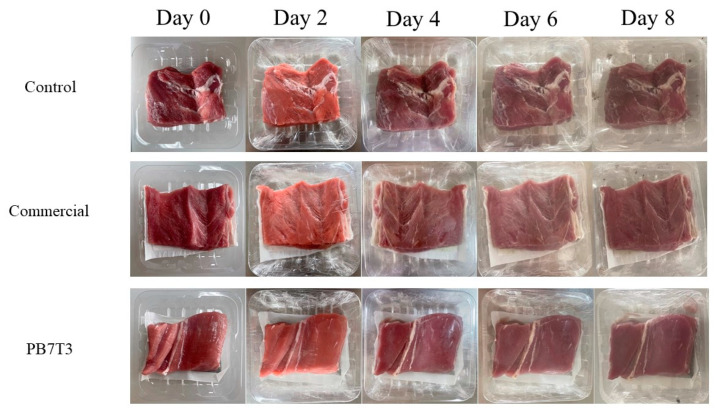
Appearance of fresh pork during storage at 4 °C.

Changes in the appearance of fresh pork during storage at 4 °C are shown in Figure 4. On the initial day of storage, all fresh pork samples displayed a purplish-red color with a firm texture, good elasticity, and a pleasant smell. As storage time progressed, the color of the pork in all treatments gradually darkened, the surface became sticky, and an unpleasant odor developed. This deterioration occurred more rapidly in the control group without an absorbent pad. By day 6, dark spots appeared on the surface of the control samples, while no such spots were observed on samples with a commercial pad or the PB7T3 pad. These results indicate that spoilage occurred more rapidly in the control group compared to pork stored with an absorbent pad.

The changes in L*, a*, and b* values of the fresh pork samples are shown in Table 2. The trend in L* values corresponded with the observed changes in appearance. Initially, the L* value was 42.67 ± 2.08, and this decreased as storage time increased. By the end of the storage period, the L* value for the control group was 36.30 ± 1.55, while the values for fresh pork treated with the commercial pad and PB7T3 were 39.47 ± 1.65 and 39.07 ± 1.10, respectively.

The a* value (redness) was initially 10.73 ± 0.64, and it decreased over time across all treatments. However, the rate of decline in the a* value was significantly faster in the control group compared to the commercial pad and the PB7T3 pad. By the end of the storage period, the a* values of the commercial pad and PB7T3 pad were 8.7% and 7.6% higher than those of the control, respectively. The decrease in a* values was attributed to microbial growth, which caused the oxidation of myoglobin to metmyoglobin, resulting in a significant reduction in the red color [25].

The increase in yellowness (b* value) in meat is attributed to lipid oxidation [25]. In this study, the b* value rose from 9.00 ± 0.20 at the start to 19.47 ± 1.62, 17.40 ± 1.25, and 16.70 ± 1.39 for the control, the commercial pad, and the PB7T3 pad, respectively. The control group showed the greatest increase in the b* value, while treatments with absorbent pads exhibited lower increases. These results suggest that the water-absorbing properties of the absorbent pads helped to reduce lipid oxidation caused by microbial growth [26].

**Figure 5:**
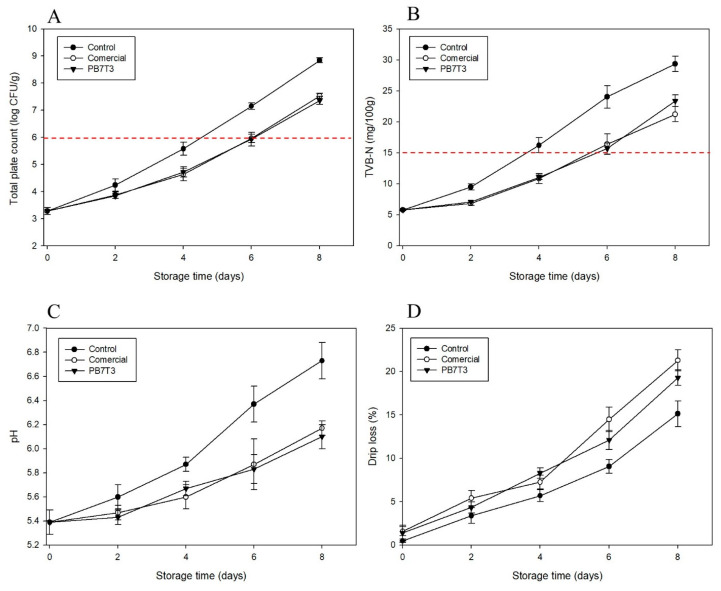
Changes in TPC (**A**), TVB-N (**B**), pH (**C**), and drip loss (**D**) during the storage of fresh pork at 4 °C.

Other indicators of pork freshness include changes in total volatile basic nitrogen (TVB-N) and pH, which are closely linked to microbial growth and protein degradation. As proteins break down, volatile nitrogen compounds are produced, leading to an increase in the pH of the meat [27,28]. The changes in the total TPC of fresh pork for all treatments are shown in Figure 5A. The initial TPC was 3.28 log CFU/g, and this increased during storage. The control group, which did not contain an absorbent pad, reached the microbial limit of 6 log CFU/g [29] after 5 days of storage. In contrast, both the commercial pad and the PB7T3 pad reached this limit on day 6. By the end of the storage period, the TPC of the control group was 8.84 log CFU/g, while the commercial pad and the PB7T3 pad had lower counts of 7.52 and 7.36 log CFU/g, respectively. The integration of an absorbent pad into fresh pork packaging delayed microbial growth and extended the shelf life of chilled pork by two days. These findings indicated that the absorbent pads effectively reduced the availability of free water by absorbing exuded liquids, thereby limiting microbial proliferation [25].

TVB-N is also a crucial indicator for assessing the freshness of chilled pork. The changes in TVB-N values during storage are illustrated in Figure 5B. The initial TVB-N of all of the pork samples was 5.77 mg/100 g, and this gradually increased with storage time. According to safety standards, the TVB-N limit for pork is 15 mg/100 g [18]. The control sample exceeded this limit on day 4, reaching 16.23 ± 1.20 mg/100 g, while the commercial pad and the PB7T3 pad reached the limit on day 6, with values of 16.40 ± 1.65 mg/100 g and 15.70 ± 0.89 mg/100 g, respectively. These results indicated that both the commercial and the PB7T3 pads significantly extended the shelf life of the pork samples.

Figure 5C presents the pH levels for all treatments. The initial pH of fresh pork was 5.39 ± 0.10, and this increased over the storage period, following a trend similar to TPC and TVB-N, as previously reported. The control sample exhibited a faster increase in pH compared to fresh pork containing absorbent pads. According to spoilage limits for fresh pork, a pH of 6.7 is designated as the spoilage point [1,27]. Consequently, the control sample was marked as spoiled when its pH reached 6.7 on day 8, while the pH values for the commercial pad and the PB7T3 on the same day were 6.17 and 6.10, respectively. The increase in pH is related to the degradation of proteins and the production of amines in pork, occurring under conditions of sufficient oxygen and nutrients. This degradation results in the formation of alkaline substances, such as ammonia and amino acids, which contribute to the rising pH value of the pork [28].

The drip loss of the fresh pork samples is shown in Figure 5D. There was a significant difference between fresh pork without an absorbent pad and pork samples with the commercial pad and the PB7T3 pad. Drip loss increased with storage time, with the highest rate observed in the commercial pad, followed by the PB7T3 pad and the control. At the end of the storage period, the drip loss percentages for the control, the commercial pad, and the PB7T3 pad were 15.13%, 21.3%, and 19.3%, respectively. Although the drip loss rate for PB7T3 was lower than for the commercial pad, the shelf life of the fresh pork was not significantly different.

## 4. Conclusions

A sustainable absorbent pad was successfully developed using PBAT/TPS films combined with hairy basil powder. The introduction of TPS into the PBAT matrix reduced the mechanical properties but increased water vapor permeability (WVP). The PBAT ratio of 70:30 demonstrated better physical properties than the 50:50 ratio, exhibiting good water absorption capacity (WAC), improved film-blowing capability, and higher yield. Consequently, the PB7T3 formulation was selected to fabricate an absorbent pad to extend the shelf life of fresh pork during cold storage. The storage results indicated that the PB7T3 absorbent pad effectively extended the shelf life of fresh pork; however, no significant difference was found compared to the commercial absorbent pad made from plastic. This research opens new avenues for developing sustainable packaging materials, contributing to reduced reliance on conventional non-biodegradable plastics.

## Figures and Tables

**Table 1:** Film characteristics.

Sample	Properties			
	Thickness (μm)	Tensile (N)	Elongation (%)	WVP (g·mm/m^2^·day·KPa)
LDPE	28.00 ± 0.90 ^c^	11.71 ± 1.13 ^b^	366.69 ± 42.88 ^c^	0.76 ± 0.04 ^d^
PBAT	28.40 ± 0.94 ^c^	29.02 ± 1.26 ^a^	595.11 ± 34.42 ^a^	2.76 ± 0.14 ^c^
PB7T3	29.80 ± 0.70 ^b^	6.07 ± 0.96 ^c^	399.77 ± 19.45 ^b^	4.89 ± 0.43 ^b^
PB5T5	32.20 ± 0.80 ^a^	2.15 ± 1.58 ^d^	60.18 ± 17.58 ^d^	5.51 ± 0.24 ^a^

Data are represented as mean ± SD (*n* = 10); ^a–d^ do not vary significantly (*p* < 0.05) with respect to each other in a column.

**Table 2:** Changes in L*, a*, and b* values of fresh pork during storage at 4 °C.

Treatment		Storage Time (Days)				
		0	2	4	6	8
L*	Control	42.67 ± 2.08 ^NS^	44.00 ± 1.53 ^NS^	41.67 ± 1.53 ^NS^	37.00 ± 1.68 ^b^	36.30 ± 1.55 ^b^
	Commercial Pad	42.67 ± 2.08 ^NS^	44.83 ± 0.29 ^NS^	41.33 ± 0.58 ^NS^	41.00 ± 1.69 ^a^	39.47 ± 1.65 ^a^
	PB7T3 Pad	42.67 ± 2.08 ^NS^	44.00 ± 1.73 ^NS^	42.00 ± 1.25 ^NS^	41.33 ± 1.53 ^a^	39.07 ± 1.10 ^a^
a*	Control	10.73 ± 0.64 ^NS^	9.50 ± 0.70 ^NS^	8.13 ± 0.32 ^NS^	7.40 ± 0.20 ^NS^	5.97 ± 0.44 ^b^
	Commercial Pad	10.73 ± 0.64 ^NS^	10.53 ± 0.76 ^NS^	9.40 ± 0.87 ^NS^	8.20 ± 0.53 ^NS^	7.19 ± 0.23 ^a^
	PB7T3 Pad	10.73 ± 0.64 ^NS^	11.23 ± 1.16 ^NS^	9.17 ± 0.58 ^NS^	8.37 ± 0.72 ^NS^	7.73 ± 0.71 ^a^
b*	Control	9.00 ± 0.20 ^NS^	11.60 ± 0.69 ^NS^	13.83 ± 0.67 ^a^	16.27 ± 0.9 ^a^	19.47 ± 1.62 ^NS^
	Commercial Pad	9.00 ± 0.20 ^NS^	10.78 ± 0.30 ^NS^	12.13 ± 0.40 ^b^	14.33 ± 1.2 ^ab^	17.40 ± 1.25 ^NS^
	PB7T3 Pad	9.00 ± 0.20 ^NS^	11.03 ± 0.72 ^NS^	12.53 ± 0.95 ^ab^	14.07 ± 0.8 ^b^	16.70 ± 1.39 ^NS^

Data are represented as mean ± SD (*n* = 10); NS, not significantly different; ^a,b^ do not vary significantly (*p* < 0.05) with respect to each other in a column.

## Data Availability

The original contributions presented in the study are included in the article. Should further inquiries necessitate additional information, the corresponding author can be contacted directly.

## Supplementary Materials

The following supporting information can be downloaded at https://www.mdpi.com/article/10.3390/foods14091525/s1, Supplemental Data S1: Film formation via extrusion blowing.
