# Supplementary figures and images for: Sustainable Absorbent Pads from Polybutylene Adipate Terephthalate/Thermoplastic Starch Films Combined with Hairy Basil (Ocimum basilicum) Powder to Enhance Meat Shelf Life
Source: Foods. 2025 Apr 26;14(9):1525. doi: 10.3390/foods14091525 (PMC12071910; doi:10.3390/foods14091525)

## Supplement data

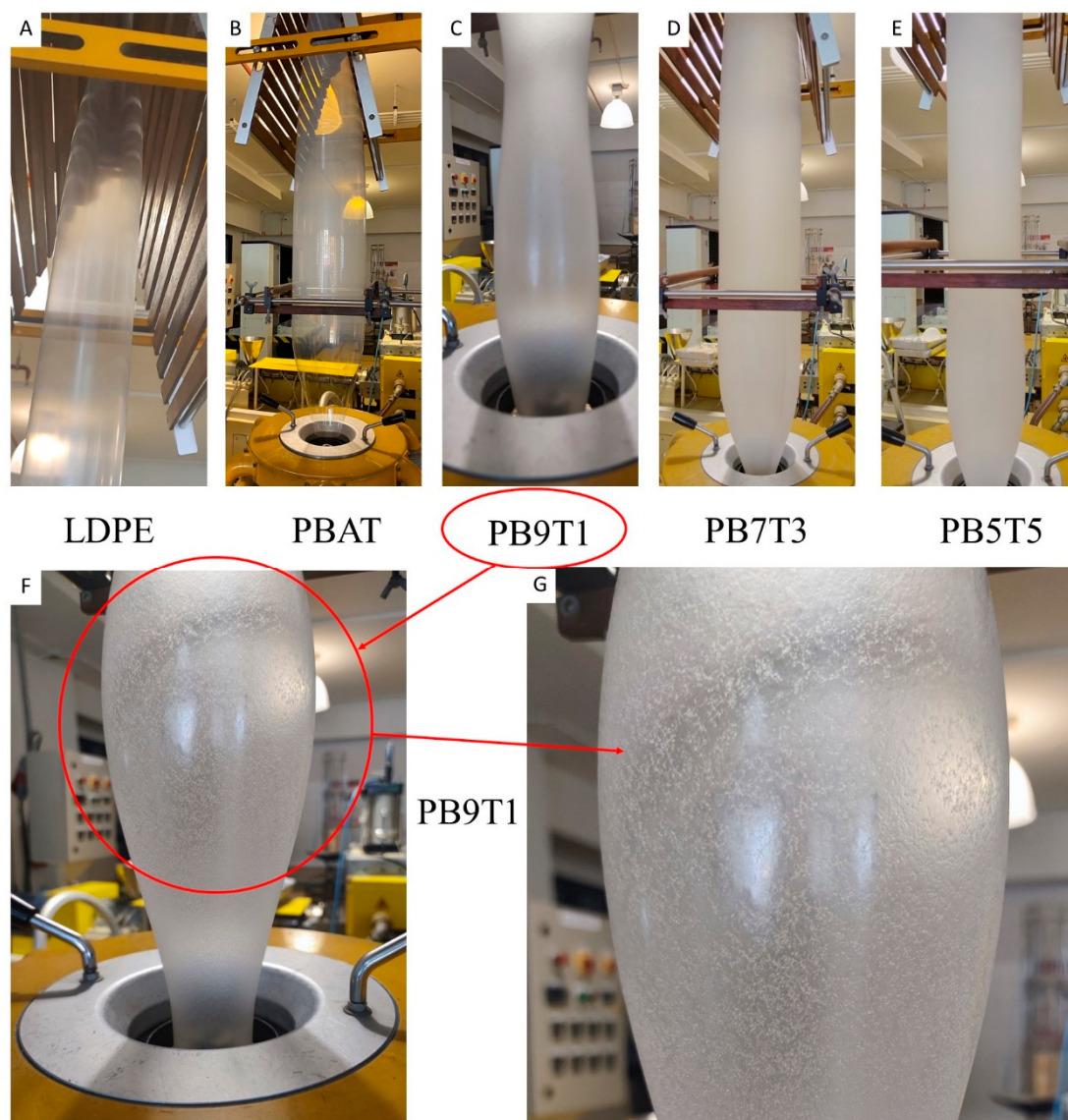

Supplement Data S1. Film formation via extrusion blowing.

Supplement: Supplementary file 1 [file foods-14-01525-s001.zip › foods-3593800-supplementary.pdf]
